# Synergistic Apoptosis Induction in Leukemic Cells by the Phosphatase Inhibitor Salubrinal and Proteasome Inhibitors

**DOI:** 10.1371/journal.pone.0004161

**Published:** 2009-01-08

**Authors:** Hannes C. A. Drexler

**Affiliations:** Max Planck Institute for Molecular Biomedicine, Department of Vascular Cell Biology, Muenster, Germany; UT MD Anderson Cancer Center, United States of America

## Abstract

**Background:**

Cells adapt to endoplasmic reticulum (ER)-stress by arresting global protein synthesis while simultaneously activating specific transcription factors and their downstream targets. These processes are mediated in part by the phosphorylation-dependent inactivation of the translation initiation factor eIF2α. Following restoration of homeostasis protein synthesis is resumed when the serine/threonine-protein phosphatase PP1 dephosphorylates and reactivates eIF2α. Proteasome inhibitors, used to treat multiple myeloma patients evoke ER-stress and apoptosis by blocking the ER-associated degradation of misfolded proteins (ERAD), however, the role of eIF2α phosphorylation in leukemic cells under conditions of proteasome inhibitor-mediated ER stress is currently unclear.

**Methodology and Principal Findings:**

Bcr-Abl-positive and negative leukemic cell lines were used to investigate the functional implications of PP1-related phosphatase activities on eIF2α phosphorylation in proteasome inhibitor-mediated ER stress and apoptosis. Rather unexpectedly, salubrinal, a recently identified PP1 inhibitor capable to protect against ER stress in various model systems, strongly synergized with proteasome inhibitors to augment apoptotic death of different leukemic cell lines. Salubrinal treatment did not affect the phosphorlyation status of eIF2α. Furthermore, the proapoptotic effect of salubrinal occurred independently from the chemical nature of the proteasome inhibitor, was recapitulated by a second unrelated phosphatase inhibitor and was unaffected by overexpression of a dominant negative eIF2α S51A variant that can not be phosphorylated. Salubrinal further aggravated ER-stress and proteotoxicity inflicted by the proteasome inhibitors on the leukemic cells since characteristic ER stress responses, such as ATF4 and CHOP synthesis, XBP1 splicing, activation of MAP kinases and eventually apoptosis were efficiently abrogated by the translational inhibitor cycloheximide.

**Conclusions:**

Although PP1 activity does not play a major role in regulating the ER stress response in leukemic cells, phosphatase signaling nevertheless significantly limits proteasome inhibitor-mediated ER-stress and apoptosis. Inclusion of specific phosphatase inhibitors might therefore represent an option to improve current proteasome inhibitor-based treatment modalities for hematological cancers.

## Introduction

In the presence of a functionally intact ubiquitin-proteasome system, newly synthesized proteins that remain unfolded in the ER, are retro-translocated back into the cytosol and immediately targeted to proteasomal degradation [1], [2]. This mechanism known as ERAD plays an important role in reducing the amount of unfolded proteins in the ER. Blocking the proteolytic activity of the proteasome by either pharmacological inhibitors such as bortezomib/PS-341 or by polyglutamine repeat containing polypeptides severely compromises ERAD, induces accumulation of misfolded proteins within the ER lumen and imposes ER stress [3]–[5].

In order to maintain ER homeostasis and eventually viability, a specific signaling circuitry has evolved in the ER, which, when engaged, is described as the unfolded protein response (UPR) [6]–[8]. By triggering this defense mechanism, cells attempt to reduce the surplus of accumulating proteins in the ER by 1. elevating the folding capacity of the ER through upregulation of ER resident chaperones, 2. by increasing the capacity of the ER-associated degradative machinery, 3. by reducing protein synthesis on a global level via curtailed translation initiation, and 4. by the translation of specific mRNAs encoding proteins involved in the regulation of redox status, amino acid metabolism and eventually cell death.

In the ER the transmembrane proteins PERK, IRE1α and ATF6 act as sentinels, which sense increasing stress and signal into the cytoplasm and nucleus [8]. Upon activation, IRE1 e.g. unleashes an intrinsic endoribonuclease activity, which leads to alternative splicing of precursor XBP1 mRNA to yield the mature XBP1 transcription factor that is required for the synthesis of ER-resident chaperones and other genes important for ER function [9]. ATF6 is eventually translocated to the Golgi, where it is proteolytically processed to become an activated transcription factor that is involved in the upregulation of XBP1 mRNA and other UPR genes [10]. PERK and related kinases in contrast phosphorylate the translation initiation factor eIF2α at a critical serine residue (Ser51) leading to inactivation of eIF2α and the subsequent global inhibition of protein synthesis [11]. In parallel, expression of the transcription factor ATF4 is selectively enhanced along with the expression of downstream target genes such as GADD34, CHOP/GADD153 and others, which participate in the control of cellular redox status and cell death [12].

The block in general protein synthesis imposed by eIF2α phosphorylation is reversed by the activity of the type I Ser/Thr specific protein phosphatase PP1a/GADD34 complex [13]. This complex apparently dephosphorylates eIF2α again when ER-homeostasis is restored and allows the cell to resume protein synthesis. Salubrinal, a low molecular weight compound, has been demonstrated to inhibit the PP1a/GADD34 complex and to protect neuronal cells against ER stress [13], probably by extending the period, in which the prolonged reduction of de-novo protein synthesis can help the cell to regain protein folding capacity, to degrade the surplus of unfolded proteins and to recover from ER stress.

Here I report that salubrinal did not protect Bcr-Abl –positive or negative leukemic cells from proteasome inhibitor-mediated ER stress and toxicity but in contrast synergistically enhanced apoptotic cell death by further boosting ER-stress, a finding, which may have impact on the future design of treatment modalities for hematological cancers.

## Materials and Methods

### Chemicals

Phosphatase inhibitors salubrinal and cantharidine were purchased from Calbiochem; sodium valproate was obtained from Sigma (Deisenhofen, Germany). Proteasome inhibitors PSI (N-carbobenzoxy-L-isoleucyl-L-g-t-butyl-L-glutamyl-L-alanyl-L-leucinal) and MG132 (N-carbobenzoxy-L-leucyl-L-leucyl-L-leucinal) were purchased from the Peptide Institute (Osaka, Japan) and bortezomib (Velcade) from Janssen-Cilag (Neuss, Germany). The caspase 3 assay was obtained from Promega, the caspase 8 substrate Acetyl-Ile-Glu-Pro-Asp-AMC (Ac-IEID-AMC) was purchased from Bachem (Heidelberg, Germany). Inhibitors and substrates (except VPA) were dissolved in DMSO as 1000× or 100× stock solutions and diluted into cell culture medium as indicated; VPA was prepared as a 2 mM stock solution in sterile water.

### Plasmids, cells and transfections

Cell lines were obtained from the German Collection of Microorganisms and Cell Cultures (DMSZ; K562 and KCL-22) or from ATCC (K562, Jurkat, HL60); MM1.S cells were a kind gift of S. Rosen. Cells were cultivated in RPMI1640 medium supplemented with 10% fetal calf serum, penicillin, streptomycin and L-glutamine. Ulf R. Rapp provided human BCL-xL in pBABEpuro; the expression vector pEF-FLAGpGKpuro containing FLAG-tagged *crmA* was a gift of D. Vaux (EMBO J. 18: 330–338 (1999)) and the ER-stress reporter constructs 5′-ATF4.GFP and CHOP::GFP were kindly provided by D. Ron. The pEF6-eIF2α-S51A-Myc-His expression vector was obtained from the BCCM/LMBP plasmid collection of the University of Ghent [14]. Transfections of K562 cells were achieved by nucleofection (Amaxa) according to the manufacturers instructions. Individual stably expressing clones were selected by transferring cells 24–48 hrs post transfection into selection medium, growth for 10–14 days and limiting dilution.

### Apoptosis Assay and determination of caspase activities

Apoptosis induction was quantified by the determination of apoptotic cells with a sub G1 DNA content as described [15], [16].

The combined caspase 3 and 7 activities were determined using the ApoOne reagent (Promega, Heidelberg) according to the manufacturers instructions. Briefly, 2.5×10^4^ K562 cells/well (96 well plate; 200 µl total volume) were challenged for 18 h with 5 nM PSI, 10 µM salubrinal and 2 mM VPA as indicated and the fluorescence signal determined at 350 nm_ex_/450 nm_em_ from 50 µl aliquots following incubation with the caspase substrate solution. Caspase-8 activities were assessed from 5×10^4^ K562 cells/well treated as described above by incubation of 100 µl cell suspension with an equal volume of assay buffer (20 mM Tris HCL pH 7.5, 100 mM NaCl, 1 mM EDTA, 10 mM DTT, 5% glycerol, 0.2% CHAPS) supplemented with 100 µM Ac-IETD-AMC as substrate for 4 hrs at 37°C and measurement of the fluorescence at 360 nm_ex_/460 nm_em_. Results were expressed as relative fluorescence units (RFU; mean±SD).

### Dose combination effects

The interaction between PSI and salubrinal was analyzed using the method by Chou and Talalay [17] and the CalcuSyn program (Biosoft, Ferguson, MO). Results from the apoptosis assays in which the sub G1 DNA content of cells had been determined were expressed as the fraction of cells affected (FA) in drug-treated versus untreated cells. A constant ratio of 1∶2000 between PSI and salubrinal was maintained when testing combinations of both drugs.

#### Cell Cycle Analysis

Cells were incubated with inhibitors as indicated for 8, 16, 24 and 36 hours and analyzed for apoptosis induction as described above. The remaining healthy cells with unfragmented chromatin (DNA content ≥2) were selected by gating and subjected to cell cycle analysis using the ModFit program (Becton Dickinson). All experiments were performed in triplicate.

### Reporter gene expression

Stably transfected K562 cells sorted for inducible ATF4.GFP or CHOP::GFP expression upon exposure to ER stress (Thapsigargin 1.5 µM, 24 hrs) were distributed onto 24 well plates (1×10^5^/1 ml) and exposed to PSI (5 nM) for 15 hrs, either alone or in combination with salubrinal (10 µM) or VPA (2 mM) respectively. During the last 30 min of the treatment CMXRos was added (100 nM, Molecular Probes) to monitor breakdown of the mitochondrial transmembrane potential. Cells were harvested by centrifugation, resuspended in PBS and immediately subjected to FACS analysis. All experiments were performed in triplicate.

### WST-Proliferation Assay

Cellular viability was assessed by the WST-1 colorimetric assay (Roche Molecular Biochemicals, Mannheim, Germany) according to the manufacturers instructions. Assays were performed on 96 well plates with 2×10^4^ K562 cells/well in triplicate with salubrinal concentrations ranging from 5–75 µM (total volume of 200 µl, 18 hrs). Untreated cells served as negative control sample.

### PP2A and PP1γ phosphatase activities

Phosphatase activities were determined on immunoprecipitates of the phosphatases. Briefly, 2×10^6^ K562 cells were treated for 18 hr with salubrinal (20 µM), PSI (10 nM), the combination of both drugs or okadaic acid (100 nM). After washing with PBS, cells were lysed for 15 min on ice either in PP1LB (for determination of PP1γ-activity; 20 mM Tris-HCl, pH 7.5, 1% Triton X-100, 10% glycerol, 132 mM NaCl, Roche complete protease inhibitor ) or in RIPA (for PP2A), supplemented with Roche complete protease inhibitor). Cell lysates containing 500 µg (PP1γ) or 300 µg (PP2A) protein were immunoprecipitated overnight at 4°C with 2–3 µg of the appropriate antibodies (anti-PP1γ: Santa Cruz sc-6108; anti-PP2A: Upstate, clone 1D6) and then incubated with Protein A-Sepharose. Immunoprecipitates were washed three times in lysis buffer, followed by resuspension in phosphatase assay buffer (PP2A: 20 mM Tris-HCl, pH7.5, 0.1 mM CaCl2; PP1γ: 50 mM Tris HCl pH 7.0, 0.2 mM MnCl2, 0.1 mM CaCl2, 125 µg/ml BSA, 0.05% Tween 20), supplemented with 100 µM 6,8-difluoro-4-methyl-umbelliferyl phosphate (DiFMUP; Invitrogen/Molecular Probes). Precipitates were allowed to react with substrate for 1 hr at 37°C on an Eppendorf Thermoshaker, centrifuged and DiFMU fluorescence was measured on a BioTek Lambda Fluoro 320 microplate reader (360 nm_Ex_/460 nm_Em_). Phosphatase activities are given as percent change relative to the control (DMSO treated cells).

### RNA extraction and RT-PCR analysis of XBP1 transcripts

XBP1 mRNA splicing was detected according to published protocols [9]. Total RNA was extracted from 3.5×10^6^ K562 cells incubated for 15 hrs with or without inhibitors by using the Illustra mini RNA isolation kit (GE Healthcare). RT-PCR for XBP1 was performed in a one-tube reaction (RobusT I, Finnzymes) with 1 µg total RNA, cDNA synthesis at 48°C for 60 min and the primers XBP1spliceF 5′-CCTTGTAGTTGAGAACCAGG-3′ and XBP1spliceR 5′-GGGGCTTGGTATATATGTGG-3′. PCR products were separated on a 2% metaphor agarose gel, which yielded a 442 bp product for unspliced and a 416 bp fragment for spliced XBP1 mRNA. A hybrid XBP1 was denominated XBP1_H_. RT-PCR for ß-actin was performed under the same conditions with primers 5′-TGTGATGGTGGGAATGGGTCAG-3′ and 5′-TTTGATGTCACGCACGATTTCC-3′, except that PCR products were separated on conventional agarose gels.

### Global inhibition of protein synthesis

Nascent proteins were labeled with the methionine analog L-azidohomoalanine (AHA) and a Click chemistry approach (Invitrogen/Molecular Probes). Briefly, cells were grown for 45 min in serum- and methionine-free medium followed by growth for 4 h in methionine-free medium supplemented with 100 µM AHA in the presence or absence of CHX (1 µg/ml) or PSI (5 nM). Following cell lysis in lysis buffer (1%SDS, 50 mM Tris HCl pH 7.5; 1 mM vanadate; 10 mM NaF; 10 mM ß-glycerophosphate; 10 mM Na_2_P_4_O_7_; 5 µM cantharidine and Roche Complete protease inhibitor cocktail) azide-labeled proteins (100 µg/sample ) were reacted with a biotin alkyne, and precipitated by addition of methanol/CHCl_3_. Air-dried pellets were then dissolved in 100 µl 2×Laemmli buffer and subjected to SDS-PAGE and western blotting. Labeled proteins were detected using horseradish peroxidase-coupled streptavidin and chemoluminescence.

### Western Blotting

Cells were washed once in PBS and lysed in RIPA buffer (150 mM NaCl, 1% Triton X100, 0.5% sodium deoxycholate, 0.1% SDS in 50 mM Tris-HCl pH 7.5) supplemented with 10 mM ß-glycerophosphate, 10 mM sodium pyrophosphate, 10 mM sodium fluoride, 1 mM sodium orthovanadate, 3 mM benzamide, 5 µM cantharidine and complete protease inhibitor cocktail (Roche, Mannheim). Insoluble debris was removed by centrifugation for 5 min at 14000 rpm in a microcentrifuge. Protein concentration of all samples was determined by a Coomassie protein assay (Pierce). Electrophoretic separations (20–50 µg protein/lane) were carried out on 10 or 12% polyacrylamide gels. Proteins were subsequently transferred to nitrocellulose membranes, membranes were blocked with TBST/4% non fat dry milk powder and incubated with primary antibodies at 4°C overnight. Primary antibodies were purchased from Alexis (caspase 8), Becton Dickinson (Bad), Biomol (PARP C2-10), Cell Signaling (cleaved caspase 3, cleaved caspase 9), Santa Cruz Biotechnology (eIF2α, phosph-eIF2α, GADD34, ATF6, Bak, Bax, Mcl-1, p21^Waf-1/Cip1^, ß-actin), Sigma (Bim, ß-tubulin, Flag M2), StressGen (KDEL), Transduction Laboratories (caspase 3, p27^Kip1^, Bcl-xL), and Zymed Laboratories (ubiquitin). Blots were developed by incubating membranes for 1 h with horseradish peroxidase conjugated secondary antibodies (Dianova) followed by enhanced chemoluminescence. Films were scanned into Photoshop (Adobe) using a flatbed scanner and adjusted for brightness and contrast.

### Statistical analysis

Where indicated statistical significance was ascertained by performing unpaired Student's T-tests. Significant differences were indicated by * (p≤0.05), ** (p≤0.01) or *** (p≤0.001); n.s. non-significant.

## Results

### Salubrinal enhanced PSI- or PSI/VPA-mediated apoptosis and cell cycle arrest of K562 cells

**Figure 1:**
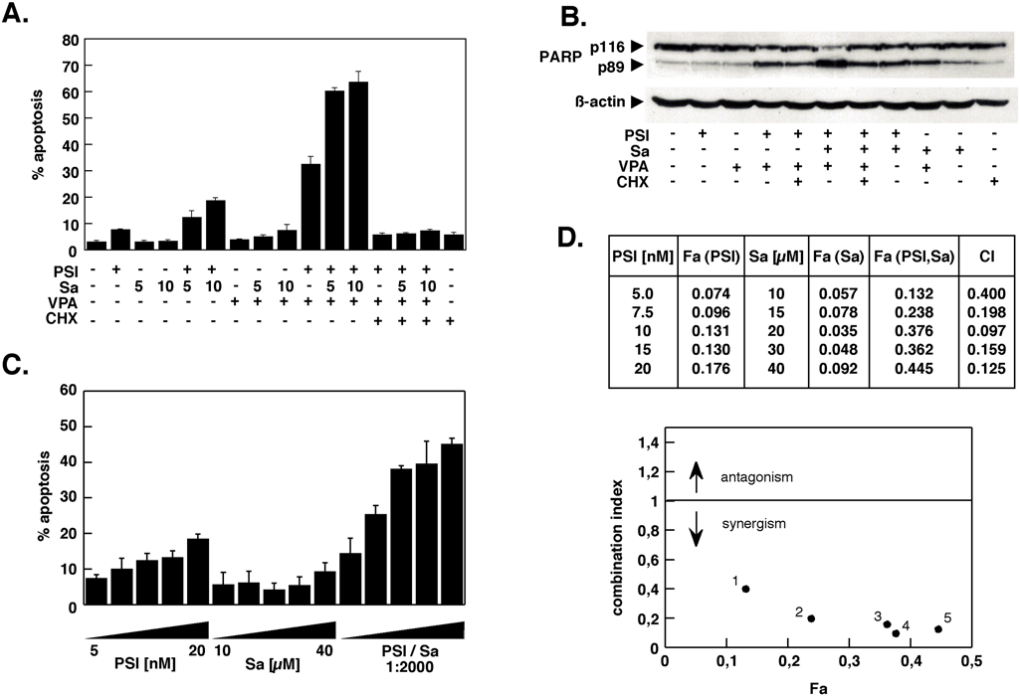
Salubrinal synergistically interacts with the proteasome inhibitor PSI to induce cell death in K562 chronic myeloid leukemia cells. (A) K562 cells were exposed to 5 nM PSI for 18 h either alone or in combination with 2 mM valproic acid and 5 or 10 µM salubrinal as indicated. The protein synthesis inhibitor cycloheximide (CHX) was used at 1 µg/ml. Apoptosis induction was assessed by propidium iodide staining and fluorescence-activated cell sorting of cells with a subdiploid (G<2N) DNA content. Results shown are the mean±SD of three determinations. (B) Whole-cell lysates were prepared from cells treated with PSI, VPA and salubrinal as in (A), separated by SDS-Page and analyzed by Western blotting for PARP-cleavage. ß-actin served as loading control. Numbers in parenthesis below the Western blot for PARP indicate the ratio of cleaved to uncleaved PARP. Shown is one representative blot obtained from at least three independent experiments with similar results. (C) K562 cells were incubated with increasing concentrations of PSI (5–20 nM), salubrinal (10–40 µM) or the combination of PSI and salubrinal at a constant ratio of 1:2000 for 18 h and apoptosis was determined as in (A). (D) Using the values obtained in (C) combination index values in relation to the fraction affected (FA) were determined by median dose effect analysis. CI values less than 1 indicate a synergistic interaction, CI values below 0.3 a strong synergism. Data are representative of at least three independent experiments, each performed in triplicate.

Treatment with proteasome inhibitors results in the generation of ER stress and the induction of apoptosis [3], [4], [18]. On the other hand salubrinal was reported to protect against ER stress [13]. It was therefore first tested whether salubrinal could protect K562 CML cells exposed to the proteasome inhibitor PSI or a combination of PSI and the histone deacetylase inhibitor VPA, which synergistically enhances the proapoptotic effect of PSI [16], [19]. HDAC inhibitors such as VPA are thought to relieve the transcriptional repression observed in various leukemic cell types that is caused by an aberrantly low level of histone acetylation and thereby prevents differentiation, cell cycle arrrest and eventually apoptosis. Unexpectedly, coadministration of salubrinal and PSI did not block or reduce the cytotoxic effect of either PSI alone or the PSI/VPA combination but further stimulated apoptosis induction under both conditions (Fig. 1A). In contrast, coadministration of salubrinal and VPA in the absence of PSI was not toxic (Fig. 1A), suggesting that salubrinal primarily boosted the effect of the proteasome inhibitor.

Induction of apoptosis was abrogated by coadministration of the translational inhibitor CHX, even when apoptosis was extensive (e.g. in the sample treated with PSI, VPA and Sa), demonstrating that cell death of K562 cells by PSI or combinations of PSI and VPA and/or salubrinal appeared to be dependent on continuous synthesis of new protein(s) and in addition correlated well with the inhibition of PARP processing (Fig. 1B), a hallmark event of apoptotic cell death.

**Figure 2:**
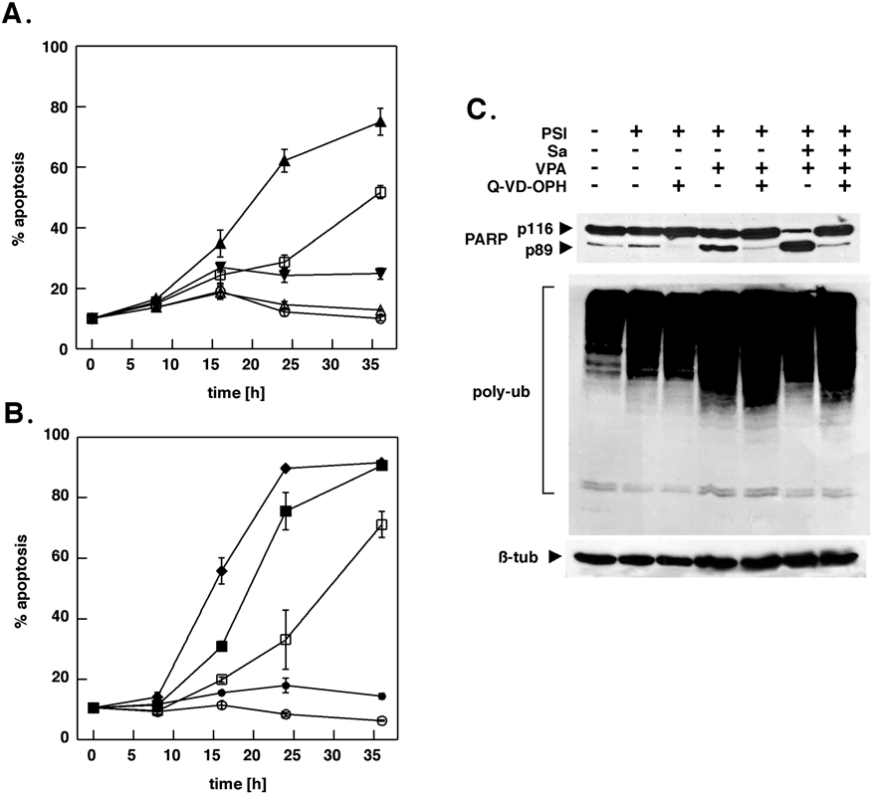
Acceleration of cell death induction by salubrinal is inhibited by the pan-caspase-inhibitor Q-VD-OPH. (A, B) K562 cells were exposed to 5 nM PSI in conjunction with 10 µM salubrinal and/or 2 mM VPA for the indicated intervals after which cells were monitored for apoptosis by FACS analysis. The pan-caspase inhibitor Q-VD-OPH was added simultaneously with the other compounds (5 µM final concentration). Data represent the means±SD of an assay performed in triplicate out of two independent experiments with similar results. DMSO control (open circles), PSI 5 nM (open squares), salubrinal 10 µM open triangles), PSI+salubrinal (filled triangles), PSI+salubrinal+Q-VD-OPH (filled inverted triangles), PSI+VPA 2 mM (filled squares), PSI+VPA+salubrinal (diamonds), PSI+VPA+salubrinal+QVD-OPH (filled circles); (C) Whole-cell lysates were prepared from cells incubated with 5 nM PSI, 2 mM VPA and 10 µM salubrinal as indicated, separated by SDS-Page and transferred to nitrocellulose membranes. The membranes were sequentially probed for PARP, polyubiquitylated proteins and ß-tubulin.

**Figure 3:**
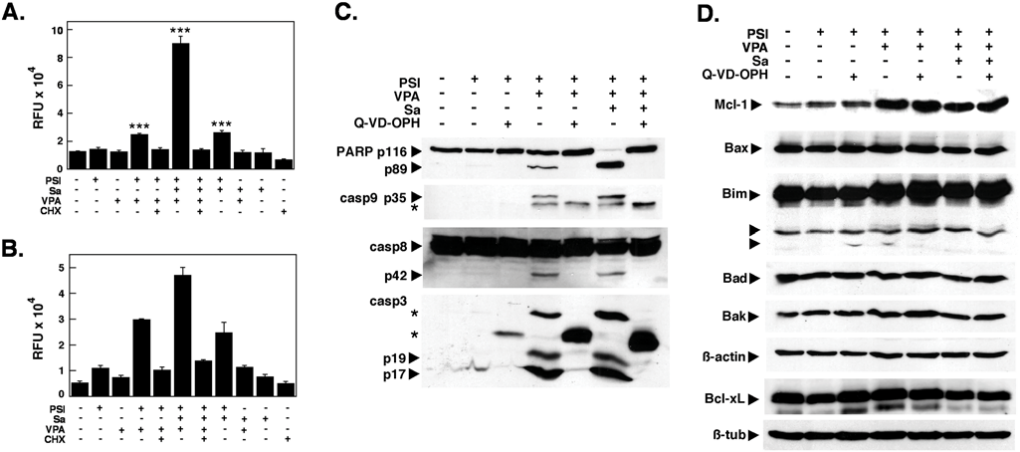
The amplification of PSI-mediated apoptosis by salubrinal is associated with activation of caspase-3 and 8 and simultaneous upregulation of Bim and Mcl-1. (A) Combined caspase 3 and 7 activities in K562 cells treated with 5 nM PSI, 10 µM salubrinal and 2 mM VPA as indicated for 18 h. (B) Caspase-8 activities in K562 cell lysates treated as in (A). Results were expressed as relative fluorescence units of the fluorescence determined at 360 nm_ex_/460 nm_em_ (RFU; mean±SD). (C) Western blot analysis. Whole-cell lysates were prepared from K562 cells treated with 5 nM PSI, 10 µM salubrinal, 2 mM VPA and 5 µM Q-VD-OPH as pan-caspase inhibitor as indicated, separated by SDS-PAGE and transferred to nitrocellulose membranes. Membranes were subsequently probed for cleaved caspase-3, 8 and 9 and for PARP. In (D) cell lysates were analyzed in an analogous fashion by sequential probing with antibodies reacting against epitopes specfic for Bim, Mcl-1, Bax, Bak, Bad and Bcl-xL. ß-actin and ß-tubulin were used as loading controls.

**Figure 4:**
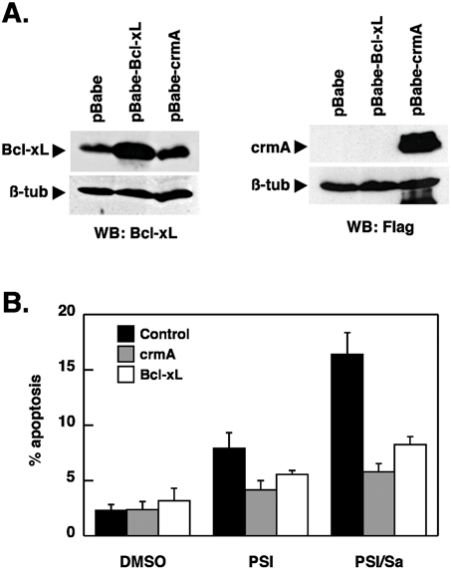
Stimulation of PSI-mediated apoptosis is delayed by overexpression of Bcl-xL and Flag-crmA. (A) Whole-cell lysates prepared from K562 cells (ATCC) stably expressing either Flag-crmA or Bcl-xL was subjected to Western blot analysis using antibodies reacting against Bcl-xL or the Flag epitope. ß-tubulin: loading control. (B) Control, Flag-crmA and Bcl-xL overexpressing K562 cells were exposed for 24 h to 20 nM PSI and 20 µM salubrinal as indicated after which apoptosis induction was determined as described in Materials and Methods (mean±SD of 3 determinations).

The increase in apoptosis was more than additive for the drug combinations containing PSI and salubrinal, raising the possibility that salubrinal and PSI could act synergistically. Therefore, a dose-effect analysis according to Chou and Talalay [17] using a constant ratio combination design was carried out (Fig. 1C,D). The calculated combination index (CI) values in fact indicated robust synergism between PSI and salubrinal (Fig. 1D). Salubrinal significantly accelerated the process of apoptosis induction by PSI (Fig. 2A) and by the combination of PSI/VPA (Fig. 2B) and could be delayed markedly by the pan-caspase inhibitor Q-VD-OPH in both instances (Fig. 2A, B). Efficient inhibition by Q-VD-OPH of apoptosis enhanced by salubrinal was also reflected by reduced levels of PARP cleavage (Fig. 2C). Western blotting experiments in addition confirmed that coadministration of salubrinal did not modulate the extend of polyubiquitination (Fig. 2C). Furthermore, caspase 3/7 (Fig. 3A) and caspase-8 (Fig. 3B) activities were significantly stimulated in cells treated with PSI/salubrinal, PSI/VPA or the PSI/VPA/salubrinal combination; while processing of these caspases and of caspase-9 was abrogated by. Q-VD-OPH (Fig. 3C).. Characteristically, Bim and Mcl-1 accumulated following treatment with proteasome inhibitor (Fig. 3D), while the levels of other members of the Bcl-2 family (Bax, Bad, Bak and Bxl-xL) remained unchanged. Finally, overexpression of Bcl-xL or crmA as demonstrated in Fig. 4A significantly delayed PSI/salubrinal-mediated cell death (Fig. 4B). In summary, theses results indicated that salubrinal effectively amplified proteasome inhibitor-mediated cytotoxicity and that death of these cells was still funneled into a caspase-dependent apoptotic pathway and was not shifted to other forms of cell death.

**Figure 5:**
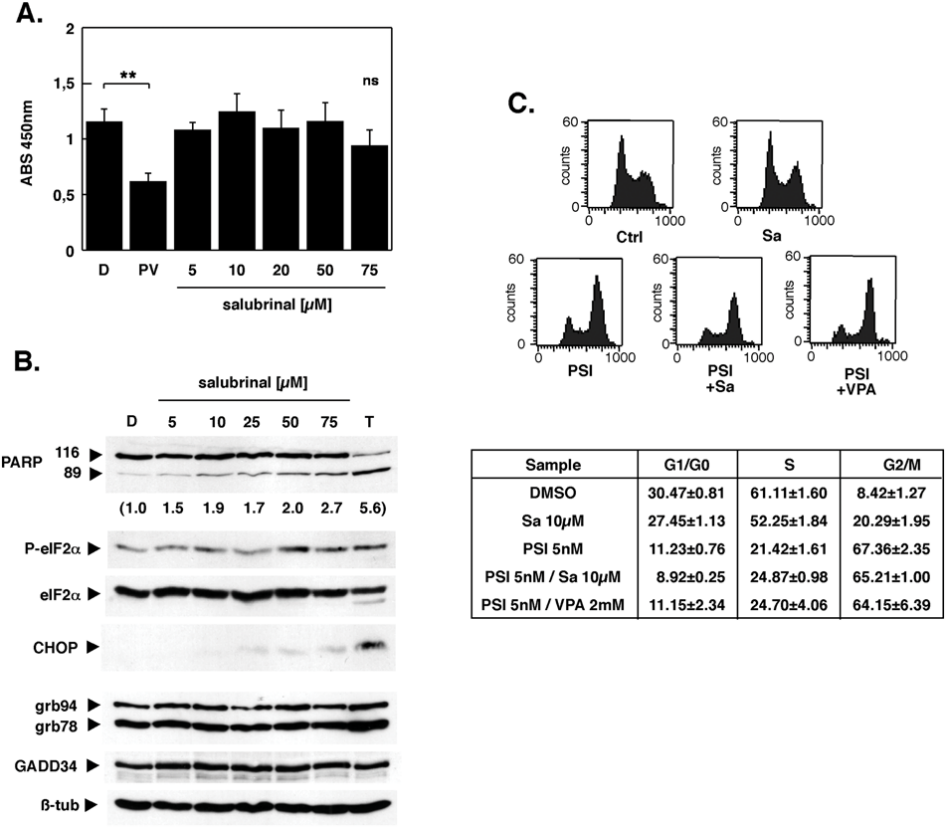
Salubrinal is nontoxic, but elicits a G2/M arrest in K562 cells. (A) K562 cells in a 96 well plate were exposed to variable concentrations of salubrinal for 15 h after which viability of cells was assessed by incubation with WST-1 reagent. Soluble formazan formation was determined by absorption measurement at 450 nm. Cells incubated with DMSO served as solvent control; incubation with 5 nM PSI and 2 mM VPA was a control for effective apoptosis induction. Data are presented as means±SD (n = 3); (B) Whole-cell lysates were prepared from cells that were treated with increasing concentrations of salubrinal, separated by SDS-PAGE and transferred to nitrocellulose membranes. Membranes were sequentially probed for P-eIF2α, eIF2α, CHOP, PARP, KDEL, GADD34 and ß-tubulin. Incubation with 2 µM thapsigargin served as positive control for the induction of ER-stress. The extent of PARP cleavage was determined by densitometry and is provided as fold increase in brakets below the Western blot for PARP. Shown are representative blots obtained from at least two independent experiments with similar results (C) K562 cells exposed to 5 nM PSI, 10 µM salubrinal and 2 mM VPA as indicated were stained with propidium iodide and analyzed by fluorescence-activated cell sorting. Histograms are representative examples. Cell cycle distribution values were derived by gating for viable cells followed by application of Modfit 3.0 software (Becton Dickinson). Data are presented as means±SD (n = 3).

Toxicity of salubrinal itself was minimal for K562 cells at the concentration used in all combination experiments, only at concentrations exceeding 50 µM was there a non-significant tendency of reduced cell viability (Fig. 5A). PARP cleavage was also minimal in the presence of salubrinal only, whereas the bona fide ER-stress inducer thapsigargin led to extensive PARP cleavage and cell death (Fig. 5B). Furthermore, salubrinal only marginally increased the phosphorylation level of eIF2α on Ser51 and the expression of downstream effectors such as CHOP, and GADD34, whereas relative protein levels of ATF6 effectors grp78/Bip and grp94 were not affected at all by salubrinal (Fig. 5B). This result showed that salubrinal at 10 µM did not affect the PP1/Gadd34 phosphatase activity and the phosphorylation status of eIF2α in K562 cells, which is in contrast to previous observations for PC12 neuronal cells, where strong and persistent phosphorylation of eIF2α by salubrinal was accompanied by the upregulation of GADD34 and CHOP for more than 36 hours [13]. To further characterize the effect of salubrinal on K562 cells, the cell cycle distribution of the viable cell population was examined. Although salubrinal increased the number of cells in G2/M phase of the cell cycle at the expense of cells in G1/G0 and S-phase (Fig. 5C), the G2/M arrest mediated by PSI was much more pronounced, confirming previous observations for PSI and other proteasome inhibitors and was not further enhanced by VPA or salubrinal (Fig. 5C). These results were also supported by western analysis of the cyclin dependent kinase inhibitors p21Cip1 and p27Kip1. Accumulation of both proteins occurred as a consequence of PSI/VPA administration, but was not further enhanced by salubrinal (data not shown). Salubrinal thus appeared to be non-toxic for K562 CML cells, but slightly impeded cell cycle progression and synergistically enhanced the cytotoxicity of the proteasome inhibitor PSI.

### Salubrinal enhanced apoptosis of proteasome inhibitors MG-132 and bortezomib

**Figure 6:**
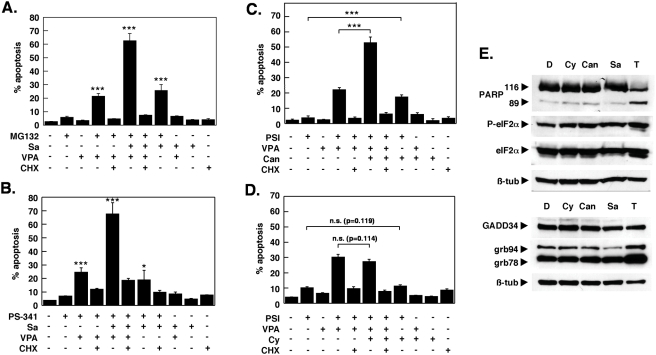
The salubrinal-enhanced toxicity is not restricted to PSI and is recapitulated by the PP1/PP2A inhibitor cantharidin. (A) K562 cells were exposed to 100 nM of the proteasome inhibitor MG132, 10 µM salubrinal and VPA for 18 h as indicated, after which apoptosis induction was assessed by fluorescence activated cell sorting of cells with a subdiploid (G<2N) DNA content. CHX was used at a concentration of 1 µg/ml. (B) K562 cells were treated as in (A), except that 5 nM bortezomib (PS-341) was used as proteasome inhibitor. (C, D) K562 cells were treated and analyzed as in (A) except that in (C) salubrinal was substituted by 0.5 µM cantharidin and in (D) by 10 µM cypermethrin. (E) Whole-cell lysates were analyzed by Western blot experiments, in which membranes were sequentially probed for P- eIF2α, eIF2α, PARP and ß-tubulin or KDEL, GADD34 and ß-tubulin. Each experimental condition was performed in triplicate, and the mean±SD from a representative experiment out of two to three independent experiments is shown.

To exclude the possibility that the observed effects of salubrinal were specific for the tetrapeptide aldehyde inhibitor PSI only, two other inhibitors, MG132 and bortezomib, were also evaluated. A nearly identical cytotoxicity profile was obtained for MG132 assayed in combination with salubrinal and/or VPA (Fig. 6A), albeit higher concentrations of MG132 were required. Similar results were also obtained when bortezomib/PS-341 was used instead of PSI (Fig. 6B), except that CHX was not as effective in reducing the extent of bortezomib-induced cell death, suggesting that bortezomib could engage slightly different signaling pathways, compared to the other two proteasome inhibitors [20]. Regardless of this possibility, salubrinal did not protect against MG132 or bortezomib/PS341 toxicity and enhanced K562 apoptosis independently from the chemical nature of the proteasome inhibitor. Furthermore, the salubrinal-mediated enhancement of proteasome inhibitor-related toxicity was not restricted to K562 cells, but was also observed in Bcr-Abl positive KCL-22 cells as well as in Bcr-Abl negative leukemic cell lines (HL-60, Jurkat, MM1.S; data not shown).

### The phosphatase inhibitor cantharidin replicated the effects of salubrinal

Since salubrinal preferentially seems to target the PP1/GADD34 complex [13], it was of interest to examine whether the effect of salubrinal could also be recapitulated by another inhibitor of this phosphatase. For this purpose cantharidin, was selected, which is less toxic than okadaic acid, but which also blocks PP1 (IC50 = 1.7 µM) activities [21]. Cypermethrin was also tested for comparison, which is a specific inhibitor of calcineurin/PP2B (IC50 = 40 pM). Neither cantharidin nor okadaic acid can inhibit activities of this phosphatase.

**Figure 7:**
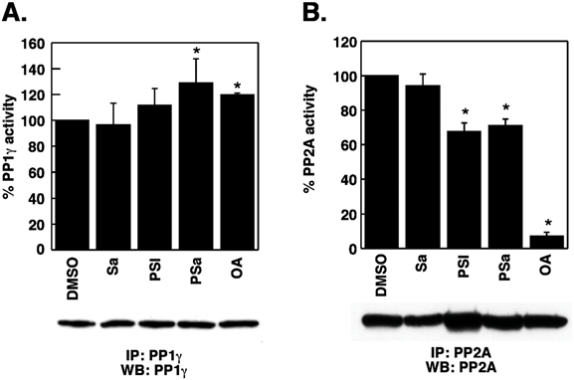
PP1γ and PP2A phosphatase activities are not affected by salubrinal. Phosphatase activities were determined on immunoprecipitates of the corresponding phosphatases as described in Materials and Methods. Following treatment phosphatases were immunoprecipitated and the activities of the corresponding phosphatases measured Phosphatase activities are given as percent change relative to the control (DMSO treated cells). Results shown are the mean±SEM of 4 independent experiments for each phosphatase.

Only the co-administration of 0.5 µM cantharidin (Fig. 6C) but not of 10 µM cypermethrin (Fig. 6D) increased the PSI or PSI/VPA-related toxicity. This concentration of cantharidin is well below the reported IC50 for growth inhibition of various tumor cell lines [22]. Like salubrinal both phosphatase inhibitors were non-toxic when applied alone at these concentrations (Fig. 6C,D) and did not notably alter phosphorylation levels of eIF2α or cause upregulation of Gadd34 (Fig. 6E). In contrast, thapsigargin (T) induced eIF2α phosphorylation at Ser51, upregulation of grp94 and grp78, extensive cell death and PARP cleavage (Fig. 6E). From these experiments it is concluded that the salubrinal-mediated increase in PSI toxicity could be recapitulated by a second phosphatase inhibitor that supposedly targets PP1. When determined from lysates of K562 cells, however, PP1 activity was not reduced in response to treatment with salubrinal, PSI or the combination of PSI and salubrinal (Fig. 7A), indicating that PP1 activity did not represent the primary target of salubrinal in K562 cells. Other phosphatases such as PP2A (Fig. 7B), PP4 and PP5 could have served as alternative targets instead [23].

### Salubrinal enhanced thapsigargin-related toxicity in K562 cells

**Figure 8:**
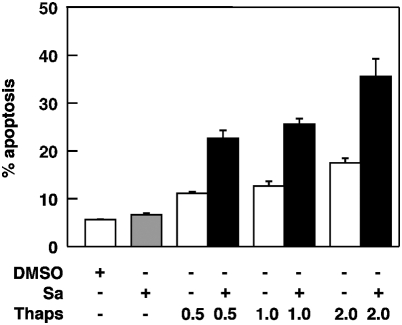
Salubrinal promotes the cytotoxic effects elicited by the ER stressor thapsigargin. K562 cells (10^5^/ml) were exposed for 18 h to thapsigargin (0.5–2.0 µM) either alone or in combination with 10 µM salubrinal. Apoptosis was determined by fluorescence activated cell sorting of cells with a subdiploid (G<2N) DNA content. Results shown represent the means±SEM of two independent experiments each performed in triplicate.

Thapsigargin, an inhibitor of the ER calcium pump, is a genuine ER stresser, capable of activating all three UPR pathways and of inducing cell death [24]. In contrast, although proteasome inhibitor treatment has been associated with ER stress and the UPR, the specific contribution of proteasome inhibitors to ER stress-mediated cell death may be obscured by the multifaceted additional impact of these inhibitors on other regulatory pathways. It was of interest therefore to determine, whether salubrinal would also prevent classical, thapsigargin-mediated ER stress mediated cell death in K562 cells or whether the response to salubrinal would instead reflect cell type specific differences. As demonstrated in Fig. 8, coadministration of salubrinal (10 µm) and of thapsigargin at low, only mildly toxic concentrations (0.5–2.0 µM) did not protect K562 cells from thapsigargin-mediated stress and toxicity and instead led to a marked increase in apoptosis. This observation suggested that the salubrinal–mediated effects were independent from the nature of the ER stressor and rather appeared to be due to intrinsic cell type specific differences in the ER stress signaling mechanisms between the leukemic cells examined here and e.g. neural PC-12 cells [13].

### Salubrinal reinforces the transcriptional activation of ATF4 and CHOP reporter genes and promotes XBP1 mRNA splicing

To further address the mechanism by which salubrinal enhances PSI mediated apoptosis, the transcriptional activation of the transcription regulator ATF4 and its proapoptotic downstream target gene CHOP were investigated. Both proteins are induced following proteasome inhibition, subsequent impairment of ERAD and the initiation of a terminal unfolded protein response [18], [25].

**Figure 9:**
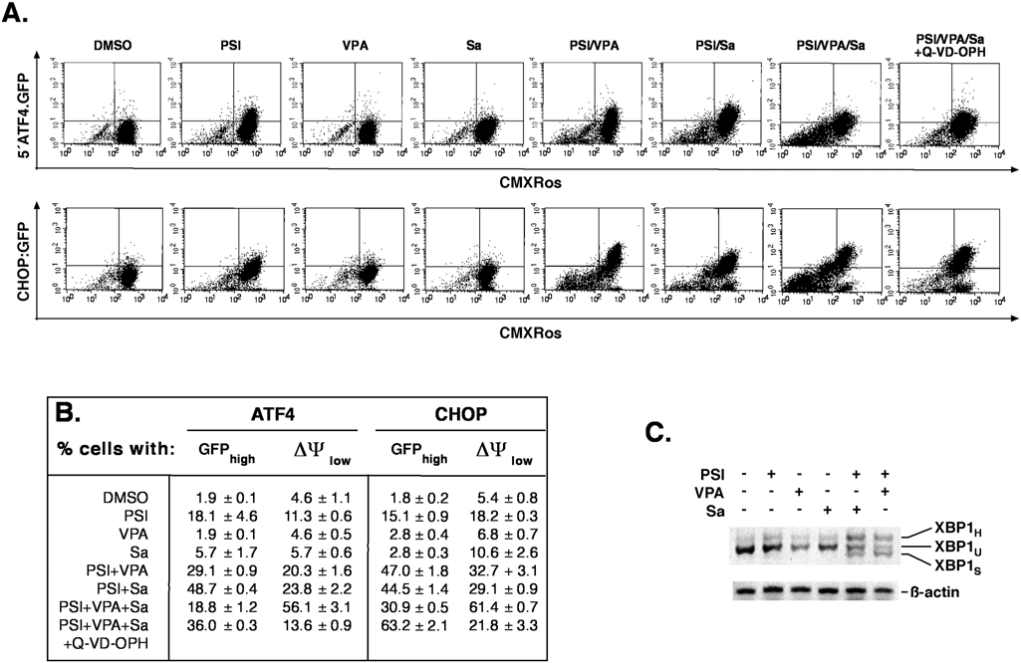
Salubrinal increases PSI-mediated transcriptional activation of ATF4 and CHOP, promotes XBP1 mRNA splicing and reduces cell viability. (A) K562 cells stably expressing a 5′ATF4.GFP or CHOP::GFP reporter gene, were challenged for 15 h with 5 nM PSI, 10 µM salubrinal and 2 mM VPA as indicated. During the last 30 min of the incubation 100 nM mitotracker orange CMXRos was added to determine mitochondrial transmembrane potential ΔΨ. Fluorescence changes were monitored by FACS analysis. The pan-caspase inhibitor Q-VD-OPH was used at a concentration of 5 µM. Scatter plots are representatives from three independent experiments each performed in triplicate with similar results and show the distribution of cells with increased GFP fluorescence, indicative for transcriptional activation of ATF4 and CHOP relative to the percentage of cells with decreased ΔΨ, indicative for reduced viability and the onset of apoptosis. (B) Numerical evaluation of results shown in (A); values represent % cells with increased GFP- and decreased CMXRos–related fluorescence, respectively. Data were obtained from one out of three independent experiment each performed in triplicate; error bars represent deviation from the mean. (C) XBP1 mRNA splicing by salubrinal and PSI. RT-PCR analysis with XBP1 or ß-actin specific primers of total RNA extracted from K562 cells, that were incubated with 5 nM PSI, 2 mM VPA and 10 µM salubrinal for 15 h as indicated. XBP_H_: hybrid XBP1; XBP1_U_: unspliced XBP1; XBP1_S_: spliced XBP1.

When exposed to ER-stress, K562 reporter cell lines expressing GFP under the control of the 5′-ATF4 or CHOP promoter [26], [27] revealed that salubrinal alone had a weak effect on 5′-ATF4 and CHOP-driven transcription of the GFP reporter when compared to DMSO-treated control cells (Fig. 9A,B). In contrast, prominent transcriptional activation of both reporter constructs was noticed upon incubation with PSI and even more pronounced, when salubrinal was combined with PSI (Fig. 9A,B). Fluorescence intensities were comparable in PSI/VPA-treated reporter cell lines. In all instances, where inhibitors provoked an increase in GFP fluorescence due to induction of an ER stress response, a substantial loss of CMXRos-positive viable cells was noted that was paralled by an increase in the fraction of (dead) cells with reduced CMXRos-related fluorescence. Loss of GFP fluorescence was apparent in cells that had received the most potent proapoptotic combination of drugs (PSI/VPA/salubrinal), possibly as a consequence of the partial loss of reporter cells due to extensive cell death. Coadminstration of the pan-caspase inhibitor Q-VD-OPH could therefore partially restore the population of viable cells with high mitochondrial DY and high levels of GFP fluorescence (Fig. 9A,B).

Activation of the transcription factor XBP1 due to alternative mRNA splicing by the activated ER resident kinase/endoribonuclease IRE1a [9] was then analyzed by RT-PCR. Using primers, which amplify characteristic fragments of each splice variant, the smaller ER-stress specific splice variant XBP1_S_ was prominent only in K562 cells treated with the PSI/salubrinal or the PSI/VPA-combination for 15 h (Fig. 9C), whereas splicing was absent (XBP1_S_) or minimal (XPB1_H_) in cells receiving the inhibitors individually (Fig. 9C).

These observations demonstrated that none of the inhibitors including salubrinal was able to activate XBP on its own. and that the combination of PSI with salubrinal or VPA was required for efficient XBP1 splicing. This result also indicated that XBP1 splicing was more closely associated with apoptosis induction than activation of ATF4, since a low dosage of proteasome inhibitor (5 nM, 15 h) that was sufficient to stimulate activation of the ATF4 and CHOP reporters (Fig. 9C) was incapable of eliciting XBP1-splicing and more extensive apoptosis (compare also with Fig. 1).

### The PSI/salubrinal-mediated apoptosis is dependent on protein translation

**Figure 10:**
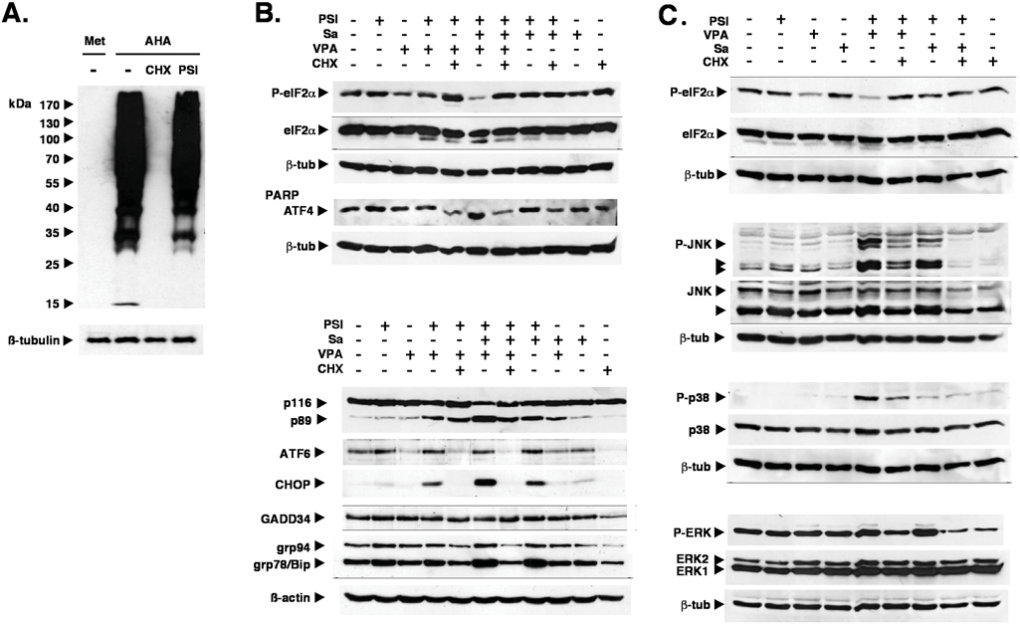
Exposure to cycloheximide (CHX) results in global inhibition of protein synthesis and abrogation of stress kinase signaling. (A) Translational inhibition by CHX in K562 cells was monitored by labeling nascent proteins with the methionine analog L-azidohomoalanine (AHA). Growth in the presence of AHA resulted in extensive labeling of proteins (lane 2), which was completely abrogated by CHX (lane 3). PSI (5 nM) slightly reduced protein synthesis (lane 4). Proteins from cells grown in the presence of methionine served as control for the labeling reaction (lane 1). Lower panel: ß-tubulin as loading control. (B, C) Whole cell lysates from K562 exposed to 5 nM PSI, 10 µM salubrinal, 2 mM VPA and 1 µg/ml CHX were subjected to SDS-PAGE and Western blot analysis as indicated. ß-tubulin or ß-actin antibodies were used to demonstrate equal loading.

Coadministration of the translational inhibitor CHX effectively inhibited apoptosis induction by PSI, MG132, bortezomib or by the combination of proteasome inhibitors with VPA and salubrinal or cantharidin (Fig. 1, 7). Direct measurements of protein synthesis revealed that CHX effectively abolished protein synthesis in K562 cells on a global level (Fig. 10A), curbing also the upregulation of ATF4 and CHOP and the ER chaperones grp78/Bip and grp94 (Fig. 10B). These results suggested that apoptosis inhibition by the translational inhibitor CHX was due to reducing the load with newly synthesized proteins, although selective suppression of the translation of pro-apoptotic factors could not be excluded.

Since ER stress conditions have also been reported to result in phosphorylation and activation of the stress kinases JNK [5], [28], [29] and p38 [8], [30], [31], it was of interest to determine, whether CHX could also reduce activation of JNK and p38 in response to PSI and salubrinal treatment. Salubrinal-enhanced PSI-mediated activation of JNK, p38 and ERK was in fact greatly diminished by coadministration of CHX (Fig. 10C). Enhanced phosphorylation of the MAPK ERK1/2 is consistent with previous reports, in which ER-stress conditions resulted in the activation of these cytoprotective kinases [32], [33], possibly to counterbalance JNK and p38 activities [34].Taken together, these observations suggested that salubrinal promoted proteasome inhibitor mediated apoptosis by exacerbating the CHX-sensitive upregulation and activation of the crucial ER-stress regulators ATF4, CHOP and XBP1 and the activation of several of their immediate downstream signal mediators. This response appeared to be largely independent from eIF2α phosphorylation and was paralleled by a reciprocal erosion of mitochondrial functionality and activation of stress kinases.

## Discussion

Ever since proteasome inhibitors have come to prominence as potent inducers of apoptosis in cancer cells and have been approved for clinical applications, it has been speculated that proteasome inhibitors kill by a mechanism unrelated to the mode of action of other more conventional chemotherapeutic drugs. Several reports have recently indicated a close correlation between the exposure of tumor cells to proteasome inhibitors, the induction of ER stress and cell death [3]–[5] and it was hypothesized that the sensitization to ER stress could represent the primary effect of proteasome inhibitors discriminating this class of inhibitors from other therapeutics. Since conflicting results have been reported regarding the role of eIF2α phosphorylation during the integrated stress response, and the series of events ultimately leading to apoptosis: [4], [18]it was of interest to analyze the role of eIF2α phosphorlyation in proteasome inhibitor-induced apoptosis of leukemic cells, by employing the recently described eIF2α dephosphorylation inhibitor salubrinal [13], [35]. Consistent with the observations made by Boyce et al., salubrinal itself was non-toxic also for K562 CML cells up to concentrations of at least 50 µM [13]. In contrast to the study by Boyce and colleagues, however, salubrinal clearly lacked a cytoprotective effect against the ER stress imposed by proteasome inhibitors and instead synergistically enhanced the cytotoxic effect of three different proteasome inhibitors in various leukemic cell lines (K562, KCL-22, HL-60, Jurkat, MM1.S). Furthermore, the observation that salubrinal also enhanced the toxic effects of thapsigargin, a bona fide ER stress inducer, excluded the possibility of inhibitor class-specific effects and instead suggested that there are intrinsic cell type specific differences in the orchestration of the PERK-eIF2α signaling cascade. Apoptosis induction by the salubrinal/PSI combination was similar in range and kinetics to a proteasome/histone deacetylase inhibitor combination such as PSI and VPA, which represents a potent stimulus for apoptosis induction in Bcr-Abl positive and negative tumor cells [16], [36]–[41] and may also trigger accumulation of unfolded proteins [42].

**Figure 11:**
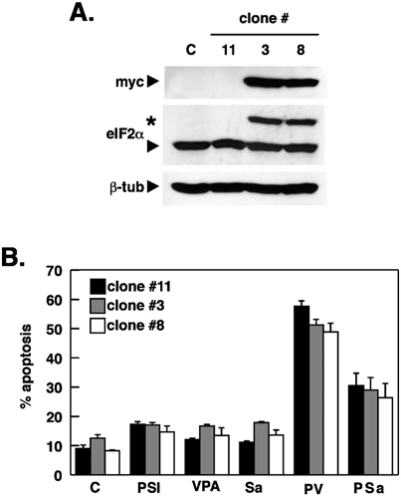
Stimulation of PSI-mediated apoptosis is not inhibited by overexpression of the transdominant eIF2α S51A mutant (A) Whole-cell lysates from K562 cells (ATCC) stably expressing myc-tagged eIF2α S51A (clone 3, 8) or a non-expressing control clone (clone 11) were subjected to Western blot analysis using antibodies reacting against the myc epitope or full-length eIF2α. ß-tubulin served as loading control. Myc-tagged eIF2α S51A is marked by an asterisk, endogenous eIF2α by an arrowhead. (B) Control and eIF2α-overexpressing K562 cells were exposed to PSI and salubrinal as indicated and the extent of apoptosis induction determined as described in Materials and Methods (mean±SD of 3 measurements).

Synergistic enhancement of PSI cytotoxicity by salubrinal was largely independent of eIF2α phosphorylation since neither salubrinal at 10 µM nor the combination of salubrinal with a proteasome inhibitor blocked PP1 phosphatase activity or led to a marked increase in eIF2α phosphorylation. This notion is also supported by the observation that substitution of salubrinal with subtoxic concentrations of the phosphatase inhibitor cantharidin induced a comparable increase in PSI-mediated cytotoxicity, whereas the PP2B/calcineurin inhibitor cypermethrin proved to be ineffective. Moreover, overexpression of a dominant-negative eIF2α S51A variant did not affect PSI/salubrinal–mediated apoptosis (Fig. 11) and upregulation of ATF and CHOP, two downstream targets of eIF2α occurred in the absence of a marked increase of eIF2α phosphorylation. These findings are not without precedent since e.g. in prostate carcinoma cells treated with PS-341, there was also accumulation of polyubiquitylated proteins and transcriptional activation of ATF4 and CHOP/GADD153 in the absence of increased phosphorylation of eIF2α [4]. The salubrinal/cantharidin-sensitive phosphatase activity nevertheless seemed to be required to maintain viability in the face of extended proteasome inhibition and when this activity was blocked, cell viability was reduced or lost. It will be interesting therefore to find out, which phosphatase exactly is affected by salubrinal, cantharidin.and similar inhibitors. PP2A, PP4 or PP5 may have to be considered as additional targets in the synergistic cooperation with proteasome inhibitors, since they all are inhibited by cantharidin, and have been implicated in contributing to apoptosis regulation [43], [44]. The synthesis of a salubrinal-derived affinity reagent may therefore be critical to pinpoint the exact molecular target of this inhibitor and to assist in shedding further light on its mode of action [35]. Identification of the phosphatase(s) targeted by salubrinal will also help to identify the corresponding phosphatase substrates and signaling pathways that are participating in survival regulation [45].

Proteasome inhibitors exert considerable cytostatic and cytotoxic effects in particular cancer cells types already as single agents, but they may be even more useful as sensitizers to apoptosis induction when delivered in combination with other anticancer drugs [16], [40], [41], [46]–[51]. Given the synergistic enhancement of proteasome inhibitor toxicity by salubrinal in K562 and other leukemic cells, salubrinal may therefore very well be added to the growing list of drugs that cooperate with proteasome inhibitor to kill hemopoietic tumor cells.

It may be speculated that cancer patients receiving proteasome inhibitor treatment could benefit from the coadministration of salubrinal also for a second reason: While enhancing the killing of sensitized leukemic cells, salubrinal may at the same time ameloriate proteasome inhibitor-mediated toxicity in neuronal cells [13], [52], [53], Saveguarding neuronal cells by this means would be a desirable feature e.g. for myeloma patients receiving proteasome inhibitor treatment, since development of peripheral neuropathy is one of the major side effects [54] and could be a direct consequence of the impairment of the ubiquitin-proteasome system [55].

Further investigations will reveal, whether salubrinal or derivatives thereoff can be included in a therapeutic strategy that is based on the induction of ER stress and maintains a strong and selective toxicity for the tumor cells on the one hand but confers protection to neuronal and other non-transformed cells on the other. These studies will have to consider also the possibility that salubrinal may exert other side effects [56], [57], due to the pleiotropic nature of phosphatase inhibitors. However, a recent proteomic study demonstrated that the number of proteins actually affected by salubrinal treatment appeared to be very limited [45], suggesting that salubrinal may possess unique features that renders it interesting enough to further develop it into a clinically useful compound.

The data presented here in summary support a paradigm shift on the protective role of the phosphatase inhibitor salubrinal during ER stress, as this compound can obviously also augment apoptosis, depending on the specific ER-stress signal and the cellular system investigated. They also suggest that the concomitant targeting of specific phosphatases in a proteasome inhibitor-based strategy to kill cancer cells could be an attractive option.
